# Accuracy of Different Indexes of Body Composition and Adiposity in Identifying Metabolic Syndrome in Adult Subjects with Prader-Willi Syndrome

**DOI:** 10.3390/jcm9061646

**Published:** 2020-05-30

**Authors:** Giorgio Radetti, Antonio Fanolla, Fiorenzo Lupi, Alessandro Sartorio, Graziano Grugni

**Affiliations:** 1 Marienklinik, Via Claudia De Medici, 2, 39100 Bolzano, Italy; 2 Observatory for Health Provincial Government, 39100 Bolzano, South Tyrol, Italy; Antonio.Fanolla@provinz.bz.it; 3 Department of Pediatrics, Regional Hospital of Bolzano, 39100 Bolzano, Italy; fiorenzolupi@gmail.com; 4 Istituto Auxologico Italiano, IRCCS, Experimental Laboratory for Auxo-endocrinological Research & Division of Auxology, 28824 Piancavallo (VB), Italy; sartorio@auxologico.it (A.S.); g.grugni@auxologico.it (G.G.)

**Keywords:** Prader-Willi syndrome, adiposity indexes, metabolic syndrome, obesity

## Abstract

(1) Objective: To compare the accuracy of different indexes of adiposity and/or body composition in identifying metabolic syndrome (MetS) in adult patients suffering from Prader‒Willi syndrome (PWS). (2) Study Design: One hundred and twenty PWS patients (69 females and 51 males), aged 29.1 ± 9.4 years, body mass index (BMI) 36.7 ± 9.9, were evaluated. The following indexes were assessed in each subject: body mass index (BMI), fat-free mass index (FFMI), fat mass index (FMI), tri-ponderal mass index (TMI), waist-to-height ratio (WtHR) and the body mass fat index (BMFI), which adjusts the BMI for the percentage of body fat and waist circumference. Thereafter, a threshold value adjusted for age and sex, which could identify MetS, was calculated for each index. (3) Results: A significant correlation was found among all indexes (*p* < 0.0001 for all). However, when the area under the curve (AUC) was compared, BMFI performed better than FMI (*p* < 0.05) and BMI better than TMI (*p* < 0.05), but only in females. (4) Conclusions: Besides small differences, all the indexes taken into consideration seem to have the same ability to identify MetS in adults with PWS. Consequently, the most easily calculated index, i.e., BMI, should be considered as the best choice. The use of thresholds appropriate for sex and age can further improve its accuracy.

## 1. Introduction

Obesity is strictly linked to metabolic syndrome (MetS), which represents a strong risk factor for atherosclerotic cardiovascular disease and type 2 diabetes mellitus (T2DM). In this context, a number of indexes have been developed, in order to define weight excess as well as body composition and visceral obesity, the latter being strongly linked with cardio-metabolic diseases [1].

Although body mass index (BMI) is the most used index in clinical practice [2], it has the limit that it is unable to discriminate fat mass from fat-free mass. Since it is well known that the percentage of fat mass and its distribution are the most important metabolic risk factors [3], other indexes have been proposed taking also into account these parameters. Among the adiposity indexes fat mass index (FMI) [4] and tri-ponderal mass index (TMI) [5] have been suggested, while waist circumference (WC) [6], model of adipose distribution (MOAD), visceral adiposity index (VAI) [7] and waist-to-height ratio (WHtR) [8] have been proposed as indexes of body fat distribution. In addition, also the fat-free mass index (FFMI) [9] has been used as a predictor of components of MetS [10], even if it reflects mainly the nutritional status in healthy and ill subjects rather than body fat or fat distribution. These surrogate indexes are routinely employed in clinical practice, since the use of expensive machinery, such as dual energy X-ray absorptiometry (DEXA) and magnetic resonance/computed tomography, is needed for the direct measurements of fat mass and visceral adiposity.

In a paper evaluating a large group of obese children and adolescents, we have recently compared different indexes of adiposity, body composition and body fat distribution, including also the body mass fat index (BMFI), which adjusts the BMI for the body composition and for the WC, in order to verify their ability in identifying MetS [11]. The main outcome of this paper was that BMI, which does not take into account body composition and fat distribution, showed the same accuracy as the other indexes, thus resulting in it being the best to be considered, also due to its easiness of calculation.

Based on these findings, we wondered whether the same results could be obtained in patients with Prader‒Willi syndrome (PWS), the most common form of syndromic obesity. PWS results from the lack of expression of the paternally-derived chromosomal region 15q11-13 [12]. These patients show neonatal failure to thrive, followed by worsening hyperphagia with early development of childhood obesity, in the absence of control [13]. PWS individuals appear to benefit from a more favorable metabolic condition in comparison to BMI-matched controls, characterized by lower insulin resistance due to the preferential subcutaneous fat distribution [14]. However, many PWS subjects develop MetS over the years, and this condition might be one of the risk factors responsible for their excessive mortality [15]. Therefore, the possibility to promptly recognize MetS or the risk of developing MetS in PWS individuals would be welcomed in order to implement more intensive treatment and prevent early mortality. In this respect, the aim of this paper was to verify which index of adiposity and/or body composition is the most accurate in identifying MetS in a large cohort of adult patients with PWS.

## 2. Patients and Methods

### 2.1. Study Population

One hundred and twenty subjects with PWS (69 females, 51 males, aged 29.1 ± 9.4 years (range 18.2–59.6), BMI: 36.7 ± 9.9 (17.2–61.6)), hospitalized in the obesity inpatient clinic of the Istituto Auxologico Italiano, Piancavallo, Verbania, Italy, were studied between January 2016 and January 2019. All but 3 males were Caucasian. All patients showed the typical PWS clinical phenotype [16]. Eighty-five subjects had interstitial deletion of the proximal long arm of chromosome 15 (del15q11-q13) (DEL15), 33 patients had uniparental maternal disomy for chromosome 15 (UPD15) and 2 individuals had a positive methylation test, but the underlying genetic defect was not identified.

At the time of the study, 24 subjects (18 females) were treated for impaired glucose tolerance (IGT: n. 4) or T2DM (n. 20): 16 with oral hypoglycemic agents alone, 1 with an oral hypoglycemic agent plus incretin mimetic, 1 with insulin alone and 6 with insulin plus metformin. Thirty-two patients (21 females) were taking monotherapy (n. 18) or a combination therapy (n. 14) for arterial hypertension. Six individuals were treated for hyperlipidemia (3 females). Twelve subjects suffered from hypothyroidism (7 females) and were adequately treated with l-thyroxine. Thirty-four females and 12 males were undergoing sex steroid replacement treatment. Behavioral abnormalities were present in all subjects, and 48 of them (23 females) were treated with neuroleptics. Twenty-four patients were on growth hormone (GH) treatment, while 54 had been treated in the past. The mean time from GH discontinuation was 11.6 ± 6.4 years (3.7–24). Forty-two had never received GH therapy.

The Ethical Committee of the Istituto Auxologico Italiano, Italy, approved the study protocol (ref. no. 01C025; acronym: PWSIPMET), and all patients and/or their parents or legal guardians gave their written informed consent to participate to the study, when appropriate. The study was performed in accordance with the Declaration of Helsinki and with the 2005 Additional Protocol to the European Convention of Human Rights and Medicine concerning Biomedical Research.

### 2.2. Anthropometric Data

Clinical examination included height, weight and WC measurements. All subjects were evaluated wearing light underwear, in fasting conditions after voiding. Anthropometry was performed by the same specifically trained operators.

Standing height was measured using a Harpenden Stadiometer (Holtain Limited, Crymych, Dyfed, UK). Body weight was measured to the nearest 0.1 kg, using standard equipment. WC was determined in standing position midway between the lowest rib and the top of the iliac crest after gentle expiration, with a non-elastic flexible tape measure.

### 2.3. Blood Pressure Measurements and Instrumental Examinations

Diastolic and systolic blood pressure (BP) were measured to the nearest 2 mmHg in the supine position after 5 min rest, using a standard mercury sphygmomanometer with appropriately sized cuff. The average of three measurements on different days was used.

Dual energy X-ray absorptiometry (DXA) was used for measurements of fat mass in kg (FM), fat mass percentage (FM%), fat-free mass in kg (FFM) and fat-free mass percentage (FFM%), using a GE-Lunar Prodigy scanner with GE Encore software version 8.80 (GE Medical Systems, Milwaukee, WI, USA) [17]. No sedation was required. All scans were performed and analyzed by the same operator.

### 2.4. Laboratory Analyses

The subjects were evaluated after a 12 h overnight fast. Parents of children with PWS were instructed to strictly supervise all food-related areas in order to avoid food consumption before testing. Baseline blood samples were drawn using venipuncture for determination of glycaemia, insulin, hemoglobin A1c (HbA1c), triglycerides (TG) and high-density lipoprotein cholesterol (HDL-C). Routine laboratory data were measured using enzymatic methods (Roche Diagnostics, Mannheim, Germany). Insulin resistance (IR) was measured using homeostasis model assessment (HOMA-IR) [18]. After exclusion of 24 PWS patients with previously diagnosed IGT/T2DM, all subjects underwent a standard oral glucose tolerance test (OGTT). Blood samples were drawn at 0, 30, 60, 90 and 120 min for measurements of plasma glucose and serum insulin levels.

### 2.5. Definitions

We considered as obese, overweight and normal-weight those subjects with a BMI >30, in the range of 25–30 and <25, respectively [2].

According to the literature [15,19], MetS is defined in the presence of three abnormal findings out of the following five parameters: central obesity, high systolic BP and/or diastolic BP, high triglycerides, low HDL-C and altered glucose metabolism (AGM). Central obesity was defined when WC was ≥94 cm for men and ≥80 cm for women [20]. Hypertension was defined in the presence of systolic BP values ≥130 mmHg and/or diastolic BP values ≥85 mmHg or in case of antihypertensive drugs use. Hypertriglyceridemia was defined in the presence of triglycerides values ≥150 mg/dl or in the case of a specific treatment. A low HDL-C level was defined with values <40 mg/dl in males and <50 mg/dl in females. Diagnosis of AGM was defined according to the American Diabetes Association criteria (IFG: fasting plasma glucose (FPG) 5.6 mmol/L to 6.9 mmol/L; IGT: 2 h PG (plasma glucose) in the 75 g OGTT 7.8 mmol/L to 11.0 mmol/L; HbA1c 5.7–6.4% (39–47 mmol/mol); T2DM: FPG levels ≥ 7.0 mmol/L or 2 h PG ≥ 11.1 mmol/L during an OGTT or HbA1c ≥ 6.5% (48 mmol/mol)] [21] or in case of antidiabetic drugs use.

Six indexes were calculated as follows:

1. BMFI [11]: BMI × FM (%) × WC (cm)
2. BMI [2]: weight (kg)/ height in m^2^
3. FFMI [9]: fat-free mass in kg/height in m^2^
4. FMI [4]: fat mass in kg/height in m^2^
5. TMI [5]: mass in kg/height in m^3^
6. WHtR [8]: WC (cm)/height (cm)

### 2.6. Statistical Analysis

The data were first scrutinized for outliers, using a cutoff of 4.5 standard deviation score. No data were excluded on this basis. To explore the data, preliminary analyses were performed. Continuous data were presented as mean (SD) or with 95% CIs. Mean values were tested for statistical significance using 2-tailed t-tests. Pearson correlation coefficients were calculated to assess the relationship between body composition indexes. Correlation analyses were used to assess the associations between each body composition index and each metabolic risk factor component. Fisher’s transformation, changing r to a Z-score, was used to compare correlated correlations.

To calculate the growth pattern of body composition indexes, a quantile regression was used [22] as an alternative to the LMS method [23]. The logarithm of each body composition index was used as response and fitted with a parametric model which involves inverse of age and square root of age. The standardized residuals were retained to represent age-adjusted values.

Receiver operating characteristic (ROC) curves were then generated to obtain the values of area under the curve (AUC) with 95% CI, and also sensitivity and specificity, for each age-adjusted standardized body composition index as predictor of MetS [24]. Assuming that BMFI should perform as well but not necessarily better than BMI, with predicted AUC values of 0.75 for BMI and 0.80 for BMFI and a non-inferiority margin of 0.10, a sample consisting of 45 adults with metabolic syndrome (MetS) and 75 without MetS allowed estimation of the non-inferiority of BMFI versus BMI (allocation ratio of 0.6). In addition, the likelihood ratio (LR+ and LR−) and positive and negative predictive values (+PV and −PV respectively) were examined.

To identify the optimal cutoff, the Youden index [25] was calculated. The corresponding percentile value for each cutoff was used in the quantile regression to identify the age-specific body composition cutoff. A median regression with the waist circumference as response and the predicted value of the body composition index at the identified percentile level was used to calculate the corresponding waist circumference for the optimal cutoff. A within-subjects ANOVA analysis was performed to compare the waist circumference means for each index within the subjects.

The significance threshold was set at *p* < 0.05. A Bonferroni test was used for multiple comparison. The data were analyzed using SAS Enterprise Guide 4.3 (SAS Institute Inc., Cary, NC, USA).

## 3. Results

According to BMI cutoffs, 35 PWS subjects were non-obese (14 normal-weight and 21 overweight, 19 females) and 85 obese (50 females). All obese subjects except 8 males had central obesity, while WC was ≥80 in 18 non-obese females and ≥94 in 12 non-obese males. Forty-three patients (24 females) had arterial hypertension. Nineteen PWS had hypertriglyceridemia (9 females), while 45 individuals had a low HDL-C level (28 females). Twenty-six subjects had T2DM (20 females), 22 had impaired glucose tolerance (IGT) (10 females) and 3 had impaired fasting glucose (IFG) (males). The presence of MetS was found in 45 subjects (28 females) (37.5%). Non-obese PWS subjects showed lower frequency of MetS (7/35: 20%) as compared with the obese group (38/85: 44.7%) (*p* < 0.05).

The clinical and laboratory characteristics of the entire study population, subdivided according to sex, are shown in Table 1.

Females showed significantly higher levels of TMI (*p* < 0.005), FMI (*p* < 0.005), HDL-C (*p* < 0.05), HbA1c (*p* < 0.05) and basal glycemia (*p* < 0.05) than males. No significant differences were found for the other parameters between the two genders.

MetS was present in 40.6% of the females and in 33.3% of the males (NS). When the study group was subdivided according to the absence/presence of MetS (Table 2), the comparison between the two genders showed higher HDL-C in females in the group without MetS (*p* < 0.005). In the group with MetS, females showed significantly higher levels of TMI, FMI and glycemia than males (*p* < 0.05), while higher basal insulin and HbA1c levels were found in males (*p* < 0.05).

The comparison of all parameters recorded in patients with and without MetS in the two genders is reported in Table 3. All the parameters related to body composition were significantly higher in patients with MetS in both genders. As expected, SBP, HbA1c, glycemia (120′) and insulin were higher in patients with MetS in both genders, while HDL-C was lower in patients with MetS. TG, DBP and glycemia (0′) were higher in females with MetS, no differences being detected in males.

### Correlations

Previous and current therapies for IGT/T2DM, arterial hypertension, hyperlipidemia, hypothyroidism, hypogonadism and GH deficiency did not influence the presence of MetS in the study group.

**Figure 1:**
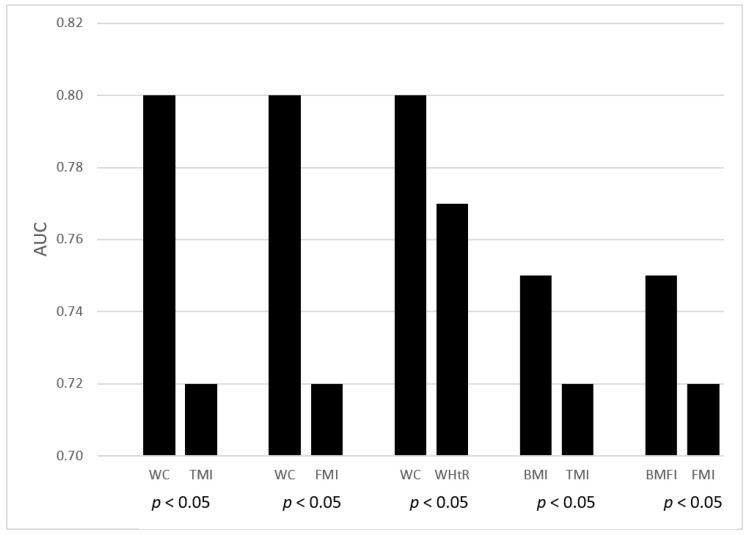
Area under curve (AUC) pairwise comparison of the different indexes of adiposity/body composition among females (only significant results are reported).

Figure 1 shows only the significant results of the AUC pairwise comparison in females, which demonstrate that BMI performed better than TMI (*p* < 0.05), BFMI better than FMI (*p* < 0.05) and WC better than TMI (*p* < 0.05), FMI (*p* < 0.05) and WHtR (*p* < 0.05). By contrast, the pairwise comparison showed comparable performances both of the remaining indexes in females and of all parameters in males.

In Table 4, the sensitivity, specificity and positive and negative predictive values for identifying the MetS, together with the likelihood ratio for each index, are reported.

The higher sensitivity was recorded for BMFI and FMI in females (*p* < 0.05) and for TMI in males (*p* < 0.05). FFMI showed the highest specificity among females, while no index performed better than the others in males.

FFMI showed the best positive predictive value among females, while no index performed better than the others among males.

BMFI and FMI showed the best negative predictive value among females, while no index performed better than the others among males.

According to the likelihood ratio, the LR+ values show that FFMI was moderately useful in identifying the presence of MetS in females, while the LR− values show that BMFI and FMI were markedly reliable in excluding MetS in females. On the other side, WC among females and TMI, FMI and BFMI among males were slightly useful in excluding MetS.

In Table S1 (Supplementary Materials) the correlations among all indexes are reported. A high statistical significance was observed for all correlations (*p* < 0.0001).

In Table S2 (Supplementary Materials) the results of the Pearson partial correlation statistics (Fisher’s z transformation), subdivided for the two genders, are shown. The Fisher’s z transformation coefficients (95% CI) are reported, showing the relative influence and the correlations of the different parameters of MetS on each adiposity index adjusted for age.

Among females, SBP, DBP and HbA1c were associated with all indexes, while basal insulin and HOMA IR were associated with WtHR and FFMI. FFMI was also associated with glycemia at 120′. Among males, HbA1c, HOMA-IR and basal insulin were associated withall indexes. SBP and DBP were associated with all indexes, apart from FFMI. TG was associated with all indexes, with the exception of FMI and TMI.

**Figure 2:**
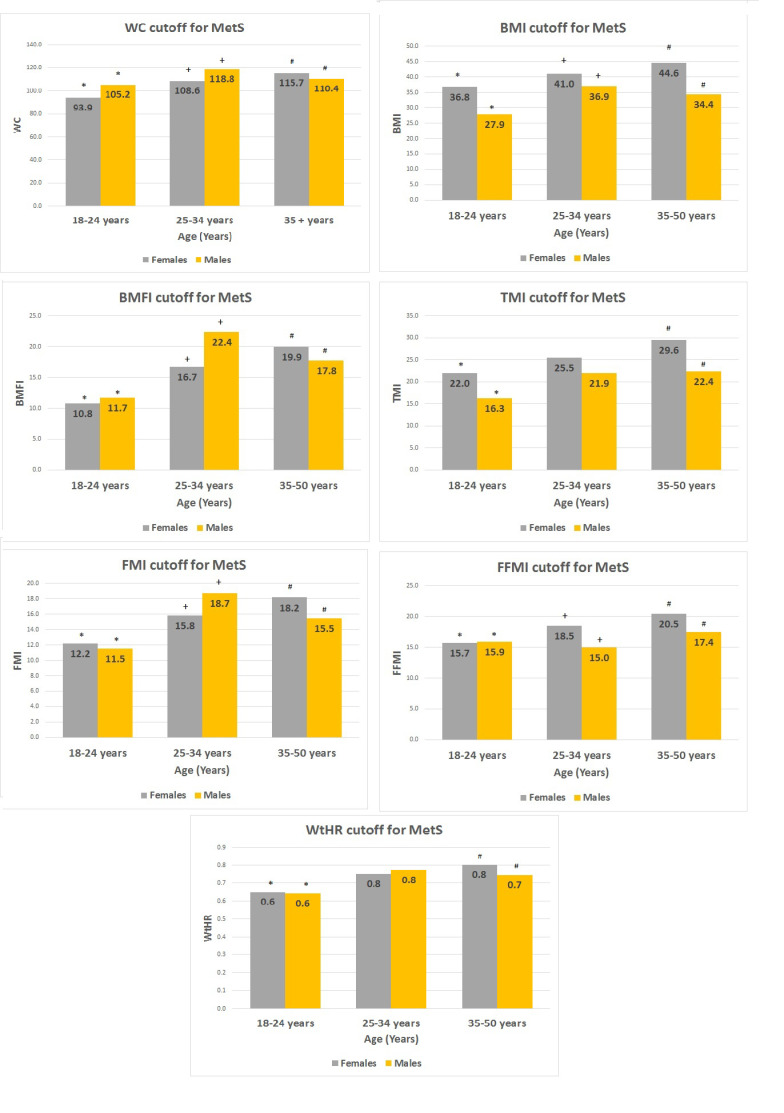
The threshold values of each index, beyond which MetS is identified, subdivided for sex and three different age groups. Within the columns, at the top, the exact threshold value is reported. * for the difference (*p* < 0.05) between 18–24 years and 25–34 years; + for the difference (*p* < 0.05) between 25–34 years and 35–50 years; # for the difference (*p* < 0.05) between 18–24 years and 35–50 years.

Finally, the threshold values of each index that identifies MetS, according to gender and different age groups, are reported in Figure 2. Females showed significant changes over the years for all indexes. The same pattern was observed in males, apart from WtHR and TMI.

## 4. Discussion

An increased mortality rate across the lifespan is present in patients with PWS compared to the general population [26]. Obesity and its comorbidities seem to be the main factors contributing to the reduced life expectancy [27], similarly to what is observed in a non-PWS population. Obesity is in fact considered the main determinant of MetS, which is specifically associated with the pandemic of cardiovascular disease and T2DM [28,29]. Despite a healthier metabolic profile in PWS, characterized by higher insulin sensitivity, we previously reported a similar prevalence of MetS in comparison to obese controls, both in children and in adults with PWS [15,30]. Therefore, it is conceivable that MetS may be responsible, at least in part, for the morbidity and early mortality in PWS adults. In this light, it is interesting to note that, adopting the same criteria of definition, the prevalence of MetS in the present study (37.5%) was not different from that observed in our previous report (34.2%) [15]. This finding suggests that despite substantial changes in the management of patients with PWS in recent years, the care of metabolic aspects has yet to be improved. This is the reason which inspired this study, with the aim to identify the most accurate index able to discover and/or predict the presence of MetS in adult PWS. The prompt recognition of this altered metabolic condition would in fact allow the implementation of measures able to prevent the long-term consequences of PWS.

The main outcome of this study is the strong correlation among all indexes (*p* < 0.0001 for all), small differences being found only in females. In fact, BMI performed better than TMI (*p* < 0.05), BFMI better than FMI (*p* < 0.05) and WC better than TMI (*p* < 0.05), FMI (*p* < 0.05) and WHtR (*p* < 0.05). On the contrary, no difference among indexes was found in males.

The NPV of all indexes were higher compared to the PPV, even if sensitivity was higher than specificity. This finding suggests that these indexes are more appropriate for excluding rather than identifying MetS.

In females, FMI and BMFI showed the highest sensitivity (*p* < 0.05), while in males TMI was the most sensitive (*p* < 0.05). FFMI showed the highest specificity among females, while in males, no index performed better than the others.

Correlation analysis showed that in both groups systolic, diastolic blood pressure and HbA1c were associated with all indexes, while for the other parameters small differences were observed, as reported in the results section. In our study no correlation was found between GH therapy and development of MetS. In this respect, it has been previously reported that GH treatment during childhood is associated with improved body composition and metabolic status in adulthood [31]. The discrepancies among the results on this topic remain to be clarified, even though they could be related both to the older age of our study group and to the longer period off therapy. In other words, we can hypothesize that the beneficial effects of GH therapy may gradually decrease over time. In addition, the small number of PWS subjects who were undergoing GH therapy during our study could explain the lack of power of some results.

The group of males with MetS had lower glucose levels than the corresponding group of females, with higher values of insulin and HbA1C. The latter, however, was in the normal range in both sexes. Although these data are not easy to interpret, they seem to indicate a more preserved pancreatic function in males, probably correlated to the lower degree of obesity in respect to females, albeit their BMI values were not statistically significant. However, this issue deserves further investigation. Finally, we were interested in verifying whether the threshold for different indexes, beyond which MetS is identified, changed according to sex and age. As illustrated in Figure 2, significant differences among age groups were observed in both genders (apart from WTHR and TMI in males), suggesting the need for appropriate thresholds in order to accurately diagnose MetS in these patients.

Previous studies, which similarly compared different adiposity indexes in the screening of MetS in the general population, showed a discriminatory power for FMI [32], WC [33] and WHtR [34]. The reason for these differences could be that only a few indexes were compared in each of the above studies, which, moreover, were cross-sectional. As a matter of fact, longitudinal studies with more indexes being compared over time are needed. Furthermore, the lack of differences between the different indexes in our study may be due to a peculiar phenotype of the PWS population, who show a different proportion of abdominal subcutaneous and visceral fat compared to BMI-matched controls [14]. Another possible explanation for these discrepancies could be the measurement of FM and FFM using bioelectrical impedance analysis [32], which is less reliable than DXA for assessing body composition.

The strength of this study is that the recruitment of PWS subjects was made in a single center and their examination was carried out by the same operators. In addition, we evaluated body composition using DXA, which represents one of the best available techniques for its evaluation. However, there are some weak points in our study. The main problem concerns the small number of patients enrolled, which results in limited strength of the statistical analysis. However, PWS is a rare disease and large experimental samples are difficult to gather. Another limitation is the lack of an appropriate control group. Nevertheless, the recruitment of such a group is almost impossible, especially patients with severe obesity. In fact, BMI-matched subjects with simple obesity are generally taller and their weight would exceed the technical capacity of DXA instruments. On the other hand, eths advise against performing DXA with normal-weight or overweight subjects. Finally, our data are derived from a transversal study. Certainly, a longitudinal evaluation would allow more information on the natural history of MetS in PWS, particularly on the time of its appearance and on its evolution. Even so, our results could be important from a rehabilitative point of view, adding value in the clinical and therapeutic management of PWS and increasing awareness of this rare pathological condition. Interestingly, our results are similar to those observed in a pediatric population of subjects with essential obesity [11], suggesting the opportunity to perform a similar investigation in both children and adolescents with PWS.

In conclusion, besides small differences, all indexes performed similarly in identifying MetS in PWS subjects, although they all had poor specificity. This suggests that from a practical point of view, BMI, which is the simplest index to be calculated, should be the preferred one, also because it does not require the evaluation of body composition and therefore leads to a reduction of the costs. Moreover, the accuracy of BMI may be further improved by using the thresholds appropriate for sex and age.

## Figures and Tables

**Table 1:** Clinical findings of Prader‒Willi syndrome (PWS) patients. For significance: * *p* < 0.05; ° *p* < 0.005.

Number of Subjects	Total	Females	Males
	120	69	51
Age yrs	29.0 ± 9.1	29.5 ± 8.3	28.3 ± 10.1
BMI	36.7 ± 9.9	38.2 ± 10.5	34.7 ± 8.9
TMI	24.1 ± 7.2	25.8 ± 7.6	21.9 ± 5.9 °
BMFI	22.2 ± 11.2	23.9 ± 11.9	19.9 ± 9.8
FMI	18.4 ± 7.0	19.9 ± 7.2	16.3 ± 6.2 °
WtHR	0.7 ± 0.1	0.7 ± 0.1	0.7 ± 0.1
FFMI	17.0 ± 3.2	16.9 ± 3.4	17.1 ± 3.0
WC (cm)	111.0 ± 18.5	110.3 ± 19.8	112.0 ± 16.7
SBP (mm/Hg)	120.3 ± 9.5	119.8 ± 9.6	121.1 ± 9.3
DBP (mm/Hg)	76.3 ± 6.0	76.1 ± 6.5	76.7 ± 5.4
HDL-C (mg/dl)	51.9 ± 14.9	54.9 ± 15.2	47.9 ± 13.7 *
TG (mg/dl)	101.2 ± 56.6	100.6 ± 65.5	101.9 ± 42.3
glycemia (mg/dl)	96.4 ± 40.3	102.8 ± 51.4	87.8 ± 11.7 *
HbA1c	5.8 ± 1.2	6.0 ± 1.5	5.5 ± 0.6 *

Abbreviations: body mass index (BMI), tri-ponderal mass index (TMI), body mass fat index (BMFI), fat mass index (FMI), waist-to-height ratio (WHtR), fat-free mass index (FFMI), waist circumference (WC), systolic blood pressure (SBP), diastolic blood pressure (DBP), high-density lipoprotein cholesterol (HDL-C), triglycerides (TG), hemoglobin A1c (HbA1c).

**Table 2:** Comparison between females and males with and without metabolic syndrome (MetS). For significance: ^&^ *p* < 0.05 for the difference vs. females; * *p* < 0.005 for the difference vs. females.

Number of Subjects	No MetS		MetS	
	Females	Males	Females	Males
	41	34	28	17
Age yr	28.1 ± 8.7	26.5 ± 9.2	31.7 ± 7.4	31.8 ± 11.1
BMI	34.2 ± 8.7	32.2 ± 9.1	44.1 ± 10.2	39.8 ± 5.7
TMI	23.1 ± 6.5	20.2 ± 6.1	29.7 ± 7.5	25.2 ± 3.8 ^&^
BMFI	19.2 ± 9.6	17.2 ± 9.9	30.9 ± 11.6	25.3 ± 7.2
FMI	17.3 ± 6.4	14.6 ± 6.4	23.6 ± 6.7	19.6 ± 4.1 ^&^
WtHR	0.7 ± 0.1	0.7 ± 0.1	0.8 ± 0.1	0.8 ± 0.1
FFMI	15.5 ± 2.3	16.4 ± 3.0	19.0 ± 3.8	18.6 ± 2.3
WC (cm)	101.3 ± 17.2	107.0 ± 15.9	124 ± 15.8	121.9 ± 14.1
SBP (mm/Hg)	116.0 ± 8.0	117.1 ± 7.3	125.4 ± 9.2	129.1 ± 7.8
DBP (mm/Hg)	74.4 ± 7.1	76.0 ± 5.5	78.6 ± 4.5	77.9 ± 5.3
HDL-C (mg/dl)	60.2 ± 14.3	50.9 ± 12.6 *	47.1 ± 13.1	41.9 ± 14.4
TG (mg/dl)	81.0 ± 34.2	94.3 ± 40.8	129.3 ± 87.3	117.2 ± 42.2
glycemia (mg/dl)	84.6 ± 11.1	85.1 ± 8.4	129.3 ± 72.4	93.2 ± 15.4 ^&^
HbA1c	5.4 ± 0.4	5.4 ± 0.5	4.4 ± 0.5	5.9 ± 0.6 ^&^
insulin	8.7 ± 5.6	9.3 ± 4.2	11.6 ± 5.3	17.4 ± 10.4 ^&^

**Table 3:** Comparison of all parameters recorded in patients with and without MetS (MetS+ vs. MetS−) in the two genders.

	Females MetS− vs. MetS+	Males MetS− vs. MetS+
BMI	*p* < 0.0001	*p* < 0.01
WC	*p* < 0.0001	*p* < 0.01
WHtR	*p* < 0.0001	*p* < 0.01
FMI	*p* < 0.001	*p* < 0.01
FFMI	*p* < 0.0001	*p* < 0.05
TMI	*p* < 0.001	*p* < 0.01
BMFI	*p* < 0.0001	*p* < 0.01
TG (mg/dl)	*p* < 0.01	n.s.
HDL-C	*p* < 0.001	*p* < 0.05
HOMA-IR	*p* < 0.01	*p* < 0.01
SBP	*p* < 0.0001	*p* < 0.0001
DBP	*p* < 0.01	n.s.
HbA1c	*p* < 0.001	*p* < 0.01
glycemia 0′	*p* < 0.01	n.s.
glycemia 120′	*p* < 0.05	*p* < 0.01
insulin 0′	*p* = 0.05	*p* < 0.01

**Table 4:** Sensitivity, specificity, positive (PPV) and negative predictive values (NPV) and the likelihood ratio (LR) for each index for identifying MetS. See the text for an explanation of the results.

	Females						Males					
	Sensitivity	Specificity	PPV	NPV	LR+	LR−	Sensitivity	Specificity	PPV	NPV	LR+	LR−
**BMI**	0.61	0.78	0.65	0.74	2.77	0.50	0.88	0.59	0.52	0.91	2.14	0.20
**TMI**	0.71	0.66	0.59	0.77	2.09	0.43	0.94	0.56	0.52	0.95	2.13	0.11
**BMFI**	0.96	0.44	0.54	0.95	1.72	0.08	0.88	0.62	0.54	0.91	2.31	0.19
**FMI**	0.96	0.39	0.52	0.94	1.58	0.09	0.88	0.65	0.56	0.92	2.50	0.18
**FFMI**	0.57	0.90	0.80	0.76	5.86	0.47	0.88	0.53	0.48	0.90	1.88	0.22
**WC**	0.89	0.61	0.61	0.89	2.29	0.18	0.82	0.56	0.48	0.86	1.87	0.32
**WtHR**	0.86	0.66	0.63	0.87	2.51	0.22	0.76	0.65	0.52	0.85	2.17	0.36

## Supplementary Materials

The following are available online at https://www.mdpi.com/2077-0383/9/6/1646/s1. Table S1: Pearson partial (age) correlation analysis. *p* < 0.0001 for all correlations. Table S2: Pearson partial correlations statistics (Fisher’z transformation). All results are shown as: Fisher’s z (95% CI).
