# Supplementary material for: Accuracy of Different Indexes of Body Composition and Adiposity in Identifying Metabolic Syndrome in Adult Subjects with Prader-Willi Syndrome
Source: J Clin Med. 2020 May 30;9(6):1646. doi: 10.3390/jcm9061646 (PMC7356766; doi:10.3390/jcm9061646)
Supplement: Supplementary file 1 [file jcm-09-01646-s001.pdf]

Table S1. Pearson partial (age) correlation analysis. $p < 0.0001$ for all correlations.

	Females, *n* = 69							Males, *n* = 51						
	**BMI**	**WC**	**WtHR**	**FMI**	**FFMI**	**TMI**	**BMFI**	**BMI**	**WC**	**WtHR**	**FMI**	**FFMI**	**TMI**	**BMFI**
**BMI**	1	0.85	0.86	0.98	0.88	0.99	0.98	1	0.89	0.89	0.97	0.79	0.99	0.97
**WC**	0.85	1	0.97	0.80	0.80	0.83	0.90	0.89	1	0.96	0.84	0.74	0.86	0.91
**WtHR**	0.86	0.97	1	0.81	0.82	0.87	0.90	0.89	0.96	1	0.86	0.68	0.91	0.91
**FMI**	0.98	0.80	0.81	1	0.77	0.96	0.97	0.97	0.84	0.86	1	0.62	0.96	0.98
**FFMI**	0.88	0.80	0.82	0.77	1	0.87	0.82	0.79	0.74	0.68	0.62	1	0.75	0.65
**TMI**	0.99	0.83	0.87	0.96	0.87	1	0.96	0.99	0.86	0.91	0.96	0.75	1	0.96
**BMFI**	0.98	0.90	0.90	0.97	0.82	0.96	1	0.97	0.91	0.91	0.98	0.65	0.96	1

Table S2. Pearson partial correlations statistics (Fisher'z transformation). All results are shown as Fisher's z (95% CI).

**Females (*n* = 69)**	**TG**	**HDL**	**HOMA-IR**	**SPB**	**DBP**	**HbA1c**	**Glycemia 0′**	**Glycemia 120′**	**Insulin 0′**
BMI	−0.1 (−0.37, 0.17)	−0.08 (−0.35, 0.19)	0.25 (−0.02, 0.49)	0.46 (0.22, 0.65) *	0.37 (0.1, 0.58) *	0.47 (0.23, 0.66) *	0.07 (−0.21, 0.34)	0.26 (−0.01, 0.5)	0.27 (0, 0.5)
WC	−0.04 (−0.31, 0.24)	−0.19 (−0.44, 0.09)	0.3 (0.03, 0.53)	0.44 (0.19, 0.64) *	0.37 (0.11, 0.59) *	0.32 (0.06, 0.55) *	0.1 (−0.17, 0.37)	0.26 (−0.01, 0.5)	0.31 (0.04, 0.54)
WtHR	−0.08 (−0.34, 0.2)	−0.09 (−0.35, 0.19)	0.28 (0.01, 0.52) *	0.43 (0.18, 0.63) *	0.32 (0.05, 0.55) *	0.31 (0.04, 0.54) *	0.11 (−0.16, 0.38)	0.25 (−0.02, 0.49)	0.29 (0.02, 0.52) *
FMI	−0.12 (−0.38, 0.16)	−0.08 (−0.35, 0.19)	0.21 (−0.06, 0.46)	0.43 (0.18, 0.63) *	0.35 (0.08, 0.57) *	0.44 (0.19, 0.64) *	0 (−0.28, 0.27)	0.2 (−0.08, 0.45)	0.24 (−0.04, 0.48)
FFMI	−0.07 (−0.34, 0.2)	−0.04 (−0.31, 0.24)	0.32 (0.06, 0.55) *	0.46 (0.21, 0.65) *	0.31 (0.04, 0.54) *	0.46 (0.21, 0.65) *	0.18 (−0.1, 0.43)	0.37 (0.11, 0.58) *	0.31 (0.05, 0.54) *
TMI	−0.12 (−0.38, 0.16)	−0.02 (−0.29, 0.25)	0.25 (−0.03, 0.49)	0.45 (0.2, 0.64) *	0.33 (0.06, 0.55) *	0.45 (0.2, 0.64) *	0.08 (−0.2, 0.35)	0.25 (−0.03, 0.49)	0.26 (−0.02, 0.5)
BMFI	−0.1 (−0.37, 0.18)	−0.12 (−0.38, 0.16)	0.23 (−0.05, 0.47)	0.46 (0.21, 0.65) *	0.37 (0.11, 0.58) *	0.41 (0.15, 0.61) *	0.02 (−0.25, 0.29)	0.21 (−0.06, 0.46)	0.25 (−0.02, 0.49)
Males (*n* = 51)									
BMI	0.3 (0.02, 0.55) *	−0.12 (−0.4, 0.18)	0.39 (0.11, 0.61) *	0.52 (0.27, 0.71) *	0.43 (0.17, 0.64) *	0.45 (0.18, 0.65) *	0.15 (−0.14, 0.42)	0.27 (−0.02, 0.52)	0.38 (0.1, 0.6) *
WC	0.38 (0.1, 0.6) *	−0.18 (−0.45, 0.12)	0.44 (0.17, 0.64) *	0.5 (0.24, 0.69) *	0.35 (0.06, 0.58) *	0.41 (0.14, 0.63) *	0.03 (−0.26, 0.32)	0.21 (−0.08, 0.47)	0.45 (0.18, 0.65) *
WtHR	0.32 (0.03, 0.56) *	−0.13 (−0.4, 0.17)	0.4 (0.12, 0.62) *	0.52 (0.27, 0.7) *	0.48 (0.22, 0.67) *	0.37 (0.09, 0.6) *	0.01 (−0.28, 0.3)	0.16 (−0.14, 0.43)	0.4 (0.13, 0.62) *
FMI	0.26 (−0.03, 0.51)	−0.08 (−0.36, 0.21)	0.36 (0.08, 0.59) *	0.53 (0.28, 0.71) *	0.5 (0.24, 0.69) *	0.4 (0.13, 0.62) *	0.18 (−0.11, 0.45)	0.25 (−0.04, 0.51)	0.34 (0.06, 0.58) *
FFMI	0.31 (0.02, 0.55) *	−0.16 (−0.43, 0.13)	0.37 (0.09, 0.6) *	0.32 (0.03, 0.56)	0.16 (−0.14, 0.43)	0.48 (0.22, 0.68) *	0.08 (−0.22, 0.36)	0.29 (0, 0.54)	0.39 (0.11, 0.61) *
TMI	0.27 (−0.03, 0.52)	−0.09 (−0.37, 0.21)	0.36 (0.08, 0.59) *	0.53 (0.29, 0.71) *	0.5 (0.25, 0.69) *	0.42 (0.15, 0.63) *	0.14 (−0.16, 0.41)	0.23 (−0.07, 0.49)	0.35 (0.07, 0.58) *
BMFI	0.29 (0, 0.54) *	−0.11 (−0.39, 0.18)	0.38 (0.1, 0.6) *	0.54 (0.3, 0.72) *	0.45 (0.18, 0.65) *	0.41 (0.14, 0.63) *	0.14 (−0.16, 0.41)	0.24 (−0.06, 0.49)	0.37 (0.08, 0.59) *

Partial variable: age. * Association between parameters of MetS and adiposity indexes.
